# Games and Telerehabilitation for Balance Impairments and Gaze Dysfunction: Protocol of a Randomized Controlled Trial

**DOI:** 10.2196/resprot.4743

**Published:** 2015-10-21

**Authors:** Tony Szturm, Jordan Hochman, Christine Wu, Lix Lisa, Karen Reimer, Beth Wonneck, Andrea Giacobbo

**Affiliations:** ^1^College of Rehabilitation SciencesDepartment of Physical TherapyUniversity of ManitobaWinnipeg, MBCanada; ^2^Otolaryngology - Head and Neck SurgeryUniversity of ManitobaWinnipeg, MBCanada; ^3^Nonlinear Systems Research LaboratoryDepartment of Mechanical and Manufacturing EngineeringUniversity of ManitobaWinnipeg, MBCanada; ^4^George and Fay Yee Centre for Healthcare InnovationDepartment of Community Health SciencesUniversity of ManitobaWinnipeg, MBCanada; ^5^Seine River PhysiotherapyWinnipeg, MBCanada

**Keywords:** balance-exercises, gaze-exercises, home therapy, telerehabilitation, therapeutic-gaming, vestibular rehabilitation

## Abstract

**Background:**

Digital media and gaming have received considerable interest from researchers and clinicians as a model for learning a broad range of complex tasks and facilitating the transfer of skills to daily life. These emerging rehabilitation technologies have the potential to improve clinical outcomes and patient participation because they are engaging, motivating, and accessible. Our research goal is to develop preventative and therapeutic point-of-care eHealth applications that will lead to equivalent or better long-term health outcomes and health care costs than existing programs. We have produced a novel computer-aided tele-rehabilitation platform that combines computer game-based exercises with tele-monitoring.

**Objective:**

Compare the therapeutic effectiveness of an in-home, game-based rehabilitation program (GRP) to standard care delivered in an outpatient physical therapy clinic on measures of balance, gaze control, dizziness, and health-related quality of life.

**Methods:**

A randomized, controlled, single-blind pilot trial will be conducted. Fifty-six participants with a diagnosis of peripheral vestibular disorder will be randomly assigned to either usual physical therapy (comparator group) or to a game-based intervention (experimental group). Measures to be assessed will include gaze control, dynamic balance, and self-reported measures of dizziness.

**Results:**

The project was funded and enrollment was started in August 2014. To date, 36 participants have been enrolled. There have been 6 drop-outs. It is expected that the study will be completed January 2016 and the first results are expected to be submitted for publication in Spring of 2016.

**Conclusions:**

A successful application of this rehabilitation program would help streamline rehabilitation services, leverage therapist time spent with clients, and permit regular practice times at the client’s convenience.

**Trial Registration:**

Clinicaltrials.gov: NCT02134444; https://clinicaltrials.gov/ct2/show/NCT02134444 (Archived by WebCite at http://www.webcitation.org/6cE18bqqY)

## Introduction

The vestibular sense organs of the inner ear are required to stabilize gaze during head motion and provide an absolute frame of reference with respect to gravity for body orientation and balance. Damage to the vestibular sense organs can lead to a number of symptoms and functional difficulties, including blurred vision, dizziness, disorientation, and falls [1]. The economic impact of vertigo and chronic imbalance is immense. Statistics from 2001 to 2004 estimated that 69 million people in the Unites States aged 40 and older report vertigo and imbalance [2-4].

The recovery of function that patients show following the damage due to peripheral vestibular lesion has long been recognized. Following peripheral vestibular lesions, sensory deprivation studies show minimal recovery [5], whereas targeted activities in enriched environments demonstrate considerable recovery of function [6-9]. A recent meta-analysis [10] demonstrated a moderate to strong evidence that a task-specific approach to vestibular rehabilitation is an effective intervention for unilateral peripheral vestibular dysfunction (PVD).

Improved patient compliance with rehabilitative care plans and increased access to these health services can improve health outcomes for individuals with chronic disabilities. In this regard, recent studies provide descriptions of the benefits of activities facilitated through video gaming. For example, studies have used the Nintendo WiiFit balance board to train and assess balance in elderly patients [11-13]. A similar approach has been developed by Szturm and colleagues [14-16] and has extended balance gaming exercises to more demanding surfaces. A recent case study of 3 individuals with PVD by Po-Yin Chen et al [17] involved adapting the Wii mote game controller to track head motion, and to use quick head movements to trigger events in a custom computer game (ie, to initiate a baseball bat to swing and hit a baseball). A preintervention and postintervention cases series was recently conducted by Szturm and colleagues [18] in 9 adults diagnosed with PVD who received a home game-based vestibular exercise program. They showed the feasibility and usability of the program. Their findings also demonstrated that using head rotation to interact with computer games, when coupled with demanding balance conditions, resulted in substantial improvements in gaze control, standing balance, and stability of the upper trunk during walking. According to the Dizziness Handicap Inventory (DHI), a self-rating, 25-item questionnaire used to quantify a participant’s perception of dizziness and its impact on quality of life, perception of dizziness decreased significantly. These findings provide preliminary support that a low-cost home game-based exercise program is well-suited to train gaze-control and balance.

## Methods

New telerehabilitation technologies (ie, digital media and innovative computer input devices) may improve clinical outcomes by making rehabilitation more motivating, accessible, and ecological. The research objectives of this study are

1. Compare the therapeutic effectiveness of a home game-based rehabilitation program to standard care delivered in an outpatient physical therapy clinic on measures of balance, gaze control, dizziness, and health-related quality of life; and

2. Examine the time course of change electronic gaze performance measures of participants in the home program.

It is postulated that the home game-based rehabilitation program will result in greater improvement in gaze control, dynamic balance control, and dizziness than a typical outpatient physical therapy regimen.

A randomized, controlled, single-blind pilot trial will be conducted (University of Manitoba Human Research Ethics Board, reference number: H2014:149).

### Recruitment

Recruitment and screening (including diagnostics) will be coordinated by physicians and physical therapists in an outpatient center for vestibular rehabilitation. Based on previous pilot research [18], we expect to be able to recruit 56 participants in 12-16 months. Our power analysis is presented in the following section. The inclusion criteria are (1) age 20-50 years, (2) diagnosis of unilateral peripheral vestibular hypofunction based on a detailed neuro-otological and neuro-orthoptic analysis that includes binocular electrooculography with caloric testing; and (3) possession of a home computer with a Windows or Mac operating system. Exclusion criteria include (1) having a central nervous system disorder (cerebrovascular accidents), multiple sclerosis, epilepsy, migraines), axial injury (fractures of the lower extremities or vertebra, advanced hip/knee osteoarthritis), and limited exercise tolerance.

### Randomization, Allocation Concealment, and Blinding

This study is designed as a randomized controlled pilot trial. Fifty-six participants will be randomly assigned to either a normative physical therapy comparator control group or to the experimental game-based exercise group. Interventions will begin within 1 week of the baseline assessment. The postintervention assessment will be conducted within 1 week of the final intervention session. Assessors will be blinded to the group assignments.

### Interventions

Current treatment for gaze instability due to loss of vestibular ocular reflex consists of rehabilitation techniques that attempt to improve gaze control by enhancing smooth pursuit, saccadic eye movements, and the optokinetic reflex; and by increasing the contribution of the remaining vestibular sensors [19-23]. For this purpose, active goal-directed activities requiring foveation, such as tracking a small visual target or reading aloud and during head movements are essential. The central nervous system must be engaged in purposeful visual fixation and exposed to graded degrees of retinal image slip (ie, head motion and object motion) [24,25]. A number of studies have used virtual reality systems with large screen displays or DVDs for optokinetic stimulation [26-28]. These optokinetic applications can provide a controlled, configurable stimulus to help clients gradually adapt to motion stimuli and visual scenes that typically induce symptoms of dizziness or disorientation. Current treatment for balance impairments includes the use of various support surfaces such as a compliant sponge pad [19,29,30]. This procedure is used to increase the magnitude and frequency of body sway in a graded fashion (analogous to use of a treadmill for gait training).

The control group will receive a standardized vestibular exercise program consisting of the Herdman gaze stabilization exercises [21,22]. This program is presently a standard of vestibular care. Participants will attend an outpatient physical therapy clinic once a week for 8 weeks. The program also includes a 2-minute home exercise program prescribed 5 times per week for a 12-week duration.

The experimental group will receive the game-based rehabilitation program delivered at home. Modern concepts of learning and neuro-adaptation have been incorporated using a task-specific approach [31,32]. This approach is similar to constraint-induced movement therapy [33], and treadmill locomotor training [34] in that it is a means of repetitive task practice and functional, goal-directed, and meaningful activities of graded intensity and complexity. Our approach will include visual fixation tasks accompanied by head and target motion, thus exposing the individual to graded degrees of retinal image slip.

Utilizing an inexpensive, commercial, motion-sensing mouse that interacts with any computer game or application, we have developed a computer-based rehabilitation platform with a therapeutic gaming application [18,35,36]. The motion-sensing mouse is a small, wireless input device with inertial sensors. These sensors and software acquire instantaneous angular position, which is then used to control the position and motion of the on-screen cursor in a manner identical to a standard computer mouse. Velcro secures the motion-sensing mouse to a headband. With this simple method, head motion is required while the participant is searching, tracking, and interacting with visual targets. This approach provides a highly flexible hands-free game-based treatment tool to train gaze control in standing position and while walking, thus incorporating graded balance demands as well as passive head motion. The use of a human interface device-compliant motion-sensing mouse allows a variety of therapeutic exercises to be coupled to a wide range of inexpensive commercial computer games. Though initially designed for clinical use, the easy-to-use and inexpensive motion-sensing mouse allows this intervention approach to be extended to home settings [18]. The use of computer games with a head rotation input device provides a simple method of graded gaze exercises consisting of visual-fixation and ocular-following tasks accompanied by head motion. We have tested and categorized more than 40 easily accessible commercial computer games. Thus, a broad range of exercise and computer game combinations are available to provide a graded rehabilitation program that includes

1. Predictable cyclic target movement with progression to random moving targets.

2. Small-amplitude movements, with progression to large-amplitude movements.

3. Slow movements, with progression to fast movements.

4. Large targets, with progression to small targets, thus requiring increased precision and foveation.

5. Solid or structured backgrounds (ie, minimal to strong optokinetic stimulation).

The amount of head motion can also be graded using a standard optical mouse and then by using the head-mounted motion-sensing mouse.

Six to eight computer games will be selected for each participant from a pool of commercial games purchased from Big Fish Games platform. Examples of computer games that will be used include the following:

1. Aquaball and Action Ball: Horizontal, single-axis brick buster with slow to moderate speed, low to moderate movement precision, a small to moderate number of distracters, and simple to complex 2D backgrounds.

2. Brave Piglet: Vertical, single-axis game play with moderate speed, moderate to high movement precision, a small to moderate number of distracters, and simple to complex backgrounds.

3. Butterfly Escape: Horizontal, single-axis matching colors with low to moderate speed, low to moderate movement precision, a small to moderate number of distracters, and simple and moving backgrounds.

4. Jet Jumper: Horizontal, single-axis driving and jumping game play with moderate to fast speed, moderate to high movement precision a medium number of distracters, and a complex and moving background.

5. Feeding Frenzy: Two-axis game play with slow motion element, low to moderate movement precision, a moderate to large number of distracters, and a moving background.

These computer games require rapid visual search, tracking of multiple objects in all directions, and active head rotations greater than 100 degrees/s. For example, many of the typical game play elements require game sprite movements covering half to full screen and head rotations up to 60 degrees. These movements are often rapid, completed in 300-500 ms. This equates to head velocities over 100 degrees/s, with precision. In general, there are four types of game objects, namely, (1) game sprite, which is controlled by head rotation movements; (2) game target objects with which to interact (sometimes more than one at a time); (3) distractor objects, which must be ignored; and (4) objects that attack the game sprite and require special attention. These game objects can be stationary or move in predictable or complex trajectories. The player needs to foveate and track multiple targets in short periods and produce head rotations to reposition game sprite with respect to game targets while also avoiding distractors or attacking objects. Usually a player’s gaze will be focused on game objects moving in a direction perpendicular or opposite to that of the game sprite or head motion. During these times, vestibular-ocular reflex (VOR) compensation will be required to maintain gaze stability. There will also be times when the eyes and head will be moving in the same direction and this will require VOR cancellation.

Each participant assigned to the experimental group will attend 3 45-minute clinical therapy sessions. Participants will receive training regarding the specific exercises and activities and use of the motion-sensing mouse and computer games. Initially the games will be played in sitting position with a standard optical hand mouse to assess the level of dizziness or nausea. The head motion mouse will be introduced in the first session. To start the exercise program, games will be selected with relatively slow target movements and with stationary backgrounds and few distractors. Game speed/amplitude, precision, number of distractors, and optokinetic features will be progressed as tolerated.

Balance training will be incorporated into the second session by playing the games while standing on a compliant sponge pad. A compliant sponge pad is now a commonly used unstable support surface in balance re-training of clients with peripheral and central nervous system disorders or older adults with a history of falls. A compliant sponge cannot completely reciprocate the normal body forces beneath the feet as the client moves. This increases the magnitude and frequency of body sway, thus increasing balance demands.

Based on the initial 3 clinical therapy sessions, a home program will be prescribed, customized to the participants’ specific balance abilities and tolerance (dizziness). Participants will be instructed to perform their exercise programs 20 minutes per day, 5 days per week. The study’s physical therapist will attend the participant’s home to set up the motion-sensing mouse and computer games, and to assess the area for fall prevention. Each participant will be instructed to use a chair to provide support. The physical therapist will email each participant weekly to monitor progress, inquire about difficulties with the computer equipment, answer questions, and progress the exercises as outlined earlier.

### Recording and Data Analysis

The following information will be collected at baseline prior to start of the interventions: age, gender, work history, history of disease/injury process, and current medications. The assessor will be blinded to participant assignments. The primary outcome measures of the study include measurement of dynamic visual acuity, balance performance, and DHI. The secondary outcome measures of the study include gaze performance and gait analysis.

#### Dynamic Visual Acuity

The test will measure the ability to see clearly during head rotations of greater than 100 degrees/s. A standardized Early Treatment Diabetic Retinopathy Study eye chart will be used, and the participants will be seated at a viewing distance of 4 m. Participants will be asked to read the letters on the eye chart, first with their head stationary and then when the head is passively rotated horizontally by the clinician/researcher at 2 Hz with the help of a metronome. The difference in the number of lines that participants are able to read when the head is stationary and when rotated will be used as the measure of dynamic visual acuity. A loss of 0-2 lines will be considered normal, whereas a loss of 3 or more lines will represent a loss of VOR function [37]. Moderate to high inter-rater and test-retest reliability has been demonstrated [38,39].

#### Balance Performance

The test protocol will consist of the following tasks performed in standing position for 45 seconds, first on a fixed floor surface and then repeated while standing on a compliant sponge surface, with eyes open and eyes closed, as described in Desai et al [40]. A force sensor array pressure-sensing mat (Vista Medical Ltd., Manitoba, Canada) will be used to compute the vertical center of foot pressure position in the anterior-posterior and medial-lateral directions. The total path length of center of foot pressure excursions will be computed and divided by total duration (45 seconds) to obtain average velocity. Increased center of foot pressure excursion and mean velocity has been interpreted as decreased stability [40,41]. A composite score will be computed, consisting of the 4 conditions of the modified Clinical Test of Sensory Interaction and Balance: eyes open and closed on fixed and sponge surfaces [29,36,42].

#### Dhi

The DHI has good test-retest reliability as well as face validity and internal consistency [43].

#### Gaze Performance

A computerized head-tracking task has been developed for testing gaze performance in standing position on fixed and sponge surfaces and during treadmill walking. Participants will be positioned on a treadmill 100 cm from an 80-cm monitor connected to a computer running the visual tracking application. For a full description and set up, see Szturm et al [18,36]. The head-tracking task involves tracking a bright visual target that moves horizontally or vertically on a computer display in a sinusoidal fashion for several cycles. Two cursors of different colors appear on the monitor. One is the reference or target cursor, which moves at a predetermined frequency of 0.4 Hz and an amplitude of 80% of the monitor width. The second cursor is user controlled and is synced with head rotation via the head-mounted motion-sensing mouse. The task requires 60 degrees of head rotation; average velocity of 50 degree/s and peak velocity of 90 degrees/s. The goal of the visual tracking task is to overlap the 2 cursors as the target cursor moves from left to right or top to bottom of the computer display. In this task, foveation is necessary for the participant to determine the amount of overlap (error) between the moving target and head cursors, and to detect when the target cursor changes direction. The computer application also generates a logged game file to synchronously record coordinates of the target cursor and head rotation at a sampling rate of 80 Hz for offline analysis of head-tracking performance described herein. The head-tracking task will be performed for 45-60 seconds under the following conditions: (1) standing on the fixed floor surface, (2) standing on a sponge surface, and (3) during treadmill walking at a speed 0.9 m/s. Before beginning the walking plus visual-tracking test, participants will walk for 5 minutes for treadmill acclimation, and to obtain baseline data of a walk-alone condition. During testing a physical therapist will stand behind each participant, ready to provide support if needed. During the treadmill walking test, the participants will wear a safety harness secured from above to a body weight support apparatus (Biodex, model, 945-482, Biodex Medical Systems, New York, USA). Body weight will not be unloaded during testing. The sponge surface and treadmill walking tasks are included to introduce varying degrees of passive head motion [44-46]. Average angular head velocities for a fixed surface has been found to be 3 degrees/s: this increases to 8 degrees/s when standing on the sponge surface and to 30 degrees/s during treadmill walking [18]. When tested on the fixed surface with minimal passive head motion, the performance of the head-tracking task would represent the function of the smooth pursuit system and also requires VOR cancellation (ie, eyes and head moving in the same direction as the target object). When passive head motion is introduced into the visual-tracking task (especially during treadmill walking), VOR compensation is also required to maintain gaze stability [47].

The coordinate data of the computer target motion and the user head rotation (motion-sensing mouse) will be used to compute gaze performance for each head-tracking task. For a full description of the data analysis methods, see Szturm et al [18,36]. A nonlinear least squares algorithm will be used to obtain a sine-wave function of the reference target cursor waveform. The first two cycles of the tracking tasks (45 seconds) will be excluded to allow participants time to acquire the moving target and begin tracking. Head rotation trajectories will be fitted to the sine-wave function, and the coefficient of determination will be computed based on the total and average residual difference between trajectories of the reference and head cursor motions. A value approaching 1 equates to perfect overlap of the two cursors, and excellent gaze performance. The coordinate data of each head-tracking trial will then be processed using custom analysis routines written in MATLAB (version 2010a; MathWorks, Natick, MA, USA).

#### Gait Analysis

A gait analysis will be conducted to examine whether the exercise program transfers to improvements in walking function. A treadmill instrumented with a pressure mat (Vista Medical, CA, USA) will be used to record vertical foot forces for each step during walking trials of 1 minute at 0.9 m/s, and thus include data for 30 consecutive steps [35,48]. The following spatiotemporal gait variables (average and coefficient of variation through 30 consecutive steps) will be computed: step and swing durations, single support times, step width, and step length.

Participants will be asked to complete daily exercise logs. The study therapist will contact each participant through phone or email on a weekly basis to obtain the exercise logs.

Interventions will begin within 1 week of the baseline assessment (primary and secondary outcome measures). The postintervention assessment will be conducted within 1 week of the final intervention session.

Participants in the experimental group will be given the visual tracking game application for home use. The game software automatically logs reference and head cursor coordinates (gaze performance) during the visual tracking task and saves the data to a coded and time-stamped computer file. Participants will be asked to play the tracking task in standing position at the beginning of every training session. This will involve 3 short tests of 45 seconds each at 3 tracking speeds. The clients can either email the data files to the investigator, or save them on a provided flash drive, which should be returned at a follow-up visit. This will allow us to perform a within individual trend analysis of up to 48 repeated measures for the experimental group.

### Statistical Analysis and Power Assessment

Descriptive statistics, including means, standard deviations, frequencies, and percentages, will be used to describe the experimental and control groups on the baseline demographic variables to achieve our first objective, which is to conduct a power analysis of the required sample size to test the difference between the experimental and controls groups at the postintervention measurement. We will test the difference between experimental and control groups on continuously and normally distributed outcome measures using analysis of covariance, with the dependent variable being the postintervention measurement of the outcome, the covariate being the preintervention measurement, and group membership being the between-participant effect. Descriptive statistics, including measures of skewness and kurtosis, will be used to assess departures from the assumptions of a normal distribution of responses. If the distribution contains extreme observations, a robust ANCOVA statistic will be adopted [49]. To test differences between experimental and control groups on discrete outcomes, such as number of times participants feel dizzy during their daily activities, we will use a Poisson regression model, with the dependent variable being the number of relevant events, the covariate being the preintervention measurement, and group membership being the between-participant effect.

To analyze the longitudinal data associated with the second objective, we will use a regression model with time as the random factor [50]. This model will be adopted because it accounts for clustering of repeated measurements within study participants and uses all available longitudinal data (ie, it does not result in case-wise deletion of study participants due to missing observations). Prior to applying this model to the data, we will conduct descriptive analyses of the proportion and pattern of missing data. To assess model fit, we will use penalized measures of the log of the likelihood function, including the Akaike Information Criterion and Bayesian-Schwarz Information Criterion. Initially we will fit a model with only a random intercept for time; subsequently, we will also consider a random slope. The intraclass correlation and the proportion of variation explained by the random effects will be calculated.

The pilot data showed a standardized effect size of .80. Assuming the number of model covariates to be 3, the proportion of variance explained by these covariates to be 10%, and a two-tailed test of the null hypothesis of no group difference at alpha=.05, we calculated that a sample size of 46 is required. Given an expected attrition rate of 20% over the study observation period for objective 2, we propose to recruit a total of 56 individuals to participate, with equal numbers for the treatment and control groups.

## Results

Enrollment for this study was started in August 2014. To date, 36 participants have been enrolled. There have been 6 drop-outs. It is expected that the study will be completed by January 2016 and the first results are expected to be submitted for publication in spring of 2016.

## Discussion

The experimental exercise program to be evaluated in this study allows different gaze, head movement, and balance exercises to be coupled with a wide range of commercial computer games. Motivation to perform tedious home programs may be improved with engaging computer games. Our platform is designed to provide client-centered and engaging programs of rehabilitation, and lead to the progression from supervised to unsupervised (monitored) home programs. This study’s findings will provide results of effectiveness and permit assessment of the potential for successful home implementation with appreciation of adverse events.

A successful application of this rehabilitation program would help streamline rehabilitation services, leverage therapist time spent with clients, and permit regular practice times at the client’s convenience. This research will address a specific, client-centered e-Health application aimed to empower individuals to manage chronic conditions and permit timely detection and intervention tools for monitoring individual and population health. The program may further assist with development of integrated solutions to support a continuum of individual and population-based care and improve accountability and access to long-term rehabilitation services.

### Limitations

With few exceptions, commercial computer games do not provide the ability to record the time played, the intensity level, or the player performance, and therefore cannot monitor the actual treatment effort. Thus, compliance will be assessed using participant self-report through completion of exercise logs. While this is common practice, the ability to have games automatically log and send actual time played, scores, and game levels achieved would assist with clinician assessment and progression of the home program.

Gaze performance is based on examination of head movements with respect to object movement. However, eye movements are not recorded and gaze position is not computed. These eye movements are required to determine the contributions of increased VOR gain and adaptations of smooth pursuit and saccadic eye movements. This study is a pre/postcase series design, and no comparisons can be made to existing vestibular rehabilitation programs.

### Conclusion

A successful application of this program would help streamline rehabilitation services, leverage therapist time spent with clients and permit regular practice times at the client’s convenience. This research will address a specific, client-centered eHealth application aimed at empowering individuals to manage chronic conditions and permit timely detection and intervention tools for monitoring individual and population health.

## Abbreviations

DHI: Dizziness Handicap Inventory
PVD: peripheral vestibular dysfunction
VOR: vestibular-ocular reflex
